# Financial and social efficiency of microcredit programs of partner organizations of Pakistan Poverty Alleviation Fund

**DOI:** 10.1371/journal.pone.0280731

**Published:** 2023-03-24

**Authors:** Zulfiqar Ali, Muhammad Asif, Naila Nazir, Ateeq Ur Rehman Irshad, Ikram Ullah, Shabir Ahmad

**Affiliations:** 1 Department of Economics, University of Peshawar, Peshawar, Pakistan; 2 Department of Statistics, University of Malakand, Lower Dir, Pakistan; 3 Department of Mathematics and General Sciences, Prince Sultan University, Riyadh, Saudi Arabia; 4 Department of Economics, University of Malakand, Lower Dir, Pakistan; 5 Department of Commerce and Management Sciences, Lower Dir, Pakistan; Marie Stopes International, PAKISTAN

## Abstract

This paper examines the financial and social efficiency of the microcredit programs offered by the Pakistan Poverty Alleviation Fund partner organizations. Panel data concerning variables of interest are collected from Pakistan Microfinance Network, covering a minimum of 14 partner organizations (in 2005) to a maximum of 35 partner organizations (in 2014). The data is analyzed using the Data Envelopment Analysis, assuming both constant and variable returns to scale scenarios and the operational scale of the partner organizations. Trends in average efficiency scores have been analyzed to assess the mission drift of the partner organizations. Results reveal that managerial inefficiency is more pronounced than the sub-optimal production scale in all three scenarios under consideration. Moreover, trends in the efficiency scores indicated a slight mission drift of the microfinance providers. About 77.5% of the partner organizations were financially sustainable over the entire study period. The study recommends providing objective-oriented training, workshops, and seminars for managing microfinance providers.

## 1. Introduction

Poverty alleviation has always remained a significant challenge, especially in the developing world. Microfinance, although a recent technology, by comparison, is considered a novel and the most effective way to alleviate poverty [1, 2]. Microfinance provides financial services such as microcredit, micro-saving, and micro-insurance to marginalized and financially deprived people with low or no access to formal financial institutions [3]. Microfinance has reportedly reduced extreme global poverty ($ 1.90 per day) from 42% in 1981 to 11% in 2013 [4]. Pakistan, realizing the potential of microfinance to uplift the socioeconomic life of its financially deprived citizens, introduced the Microfinance Ordinance [5] and Poverty Reduction Strategy paper in 2001 [6]. Likewise, the State Bank of Pakistan (SBP) is the first in the world to have introduced a policy framework regarding Micro Finance Providers (MFPs) [7].

In Pakistan, most microfinance institutions work under the Pakistan Poverty Alleviation Fund (PPAF) as Partner Organizations (POs). The primary funding bodies of the PPAF are the World Bank, the German Development Bank, and the Government of Pakistan [8]. Thus, PPAF is a funding, implementing, and monitoring agency that operates through its POs to implement various community-driven projects. The PPAF is a major stakeholder in the microfinance market since it holds a market share of more than 44% with 130 POs and has geographical coverage in more than 129 districts. [9].

However, the microfinance experience in Pakistan is not as successful as in the rest of the world [10]. According to the Microfinance Strategy Paper (2007), the country’s target was to reach more than three million borrowers by the end of 2010, which was expected to reach ten million people by the end of 2015 [11]. However, only 2.34 million borrowers were served by MFPs till the end of the fiscal year 2012–13 [12]. Besides other things, one of the core attributes that may have caused divergent results from similar technology could be the level of efficiency of the microfinance institutions in Pakistan as compared to the rest of the world. In 2015 a report from the State Bank of Pakistan confirmed that the Operational Self-sufficiency (OSS) of the MFPs in Pakistan is merely 94%, compared to 109%-118% in the rest of the world [13].

In the framework of microfinance, efficiency refers to the efficient utilization of inputs such as human & physical capital owned by MFPs to produce maximum output measurable in terms of loan portfolio & number of active borrowers [14]. Given the dual role of MFPs, efficiency is usually decomposed into social and financial efficiency. This simultaneity between the socioeconomic efficiency of MFPs differentiated them from conventional financial institutions [10]. Therefore, an assessment of the performance of the MFPs involves both its financial viability and social outreach, whereby social outreach refers to "the social value of the output of a microfinance organization in terms of depth, worth to users, the cost to users, breadth, length, and scope" [15]. On the other hand, financial viability measures sustainability, which can eventually provide funds to MFPs. [16]. To understand true status, MFPs should be analyzed from the perspectives of both social as well as financial [17, 18]

Furthermore, unlike conventional financial institutions, MFPs receive deposits, donations, and grants. The donors want to evaluate not only the economic but also the social performance of the MFPs. The MFPs are, therefore, under continuous pressure to perform their social and financial roles [19]. To understand the true value of MFPs, have suggested that the social outputs of MFPs should be studied from the perspective of social efficiency [20]. Social and financial efficiency assessments are also helpful for optimal policy agendas [10]. The MFPs are desired to provide an optimum number of social outputs compatible with the available resources. Hence, the current study is designed to investigate the social & financial efficiency of the microcredit programs under PPAF through its POs. The rest of the paper is organized as follows. Section 2 outlines the theoretical framework of the study. Section 3 presents the materials and methods used in the study. Section 4 contains results of the study with subsequent discussion. Section 5 concludes the paper and recommends some guidelines for future research.

### 2. Theoretical framework of the study

A poor person may be conceptualized as having all the abilities to earn a living except critical financial capital [21, 22]. Conceptualizing poverty in such terms thus assumes that poor people are more productive, can profitably run their small businesses, and are creditworthy enough to return their loans in due time. In the absence of collateral to obtain credit from the conventional financial institution, poor people heavily rely on informal credit sources [23], which, in most cases, results in their exploitation [24]. Hence, the realization that the poor are otherwise productive and creditworthy people but are exploited by local money lenders in the absence of formal credit availability resulted in the emergence of microfinance [25].

**Fig 1:**
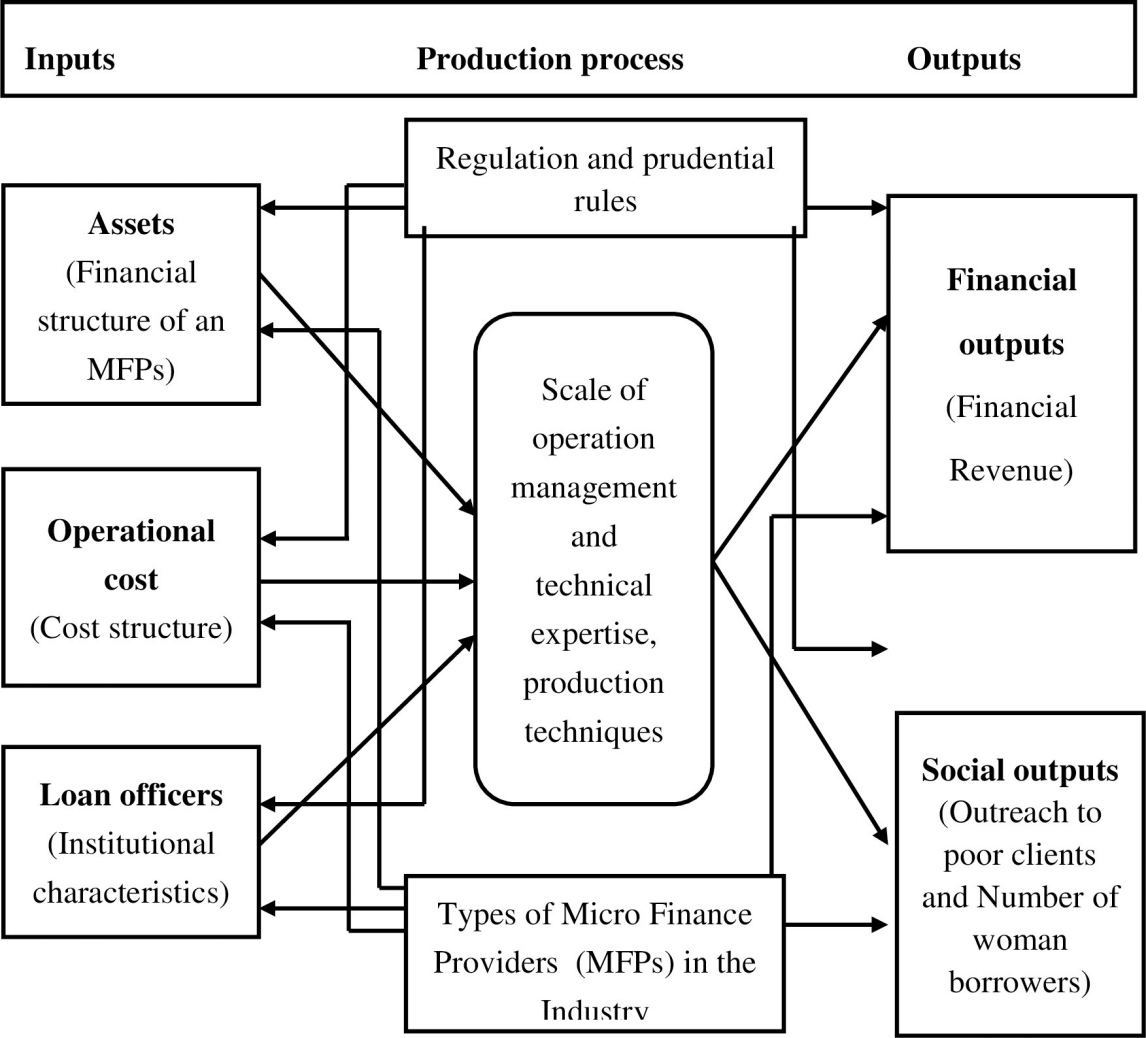
Theoretical framework of the study.

Neoclassical economics assumes an efficient production process, both technically and economically [26]. Technical efficiency means the optimum utilization of resources without wastage. Economic efficiency, on the other hand, refers to solving optimization problems involving prices. Variations in the production of various firms may be caused by technological differences, efficiency levels in production processes, and the environment where production occurs. Since microfinance follows almost the same production technology worldwide, the present study evaluates the performance of the POs of PPAF through social and financial efficiency measures. The theoretical framework of the study is explained in Fig 1.

## 3 Research methodology

### 3.1 The data

The data were collected from the annual reports of the Pakistan Microfinance Network (PMN) from 2005 to 2015. The data were stored in an excel sheet and are available as supplementary material. As mentioned earlier, 130 POs operating under PPAF, but the study considered only those POs operating for more than five years in Pakistan. The number of POs in the selected sample varies from year to year, ranging from a minimum of 14 (in 2005) to a maximum of 35 (in 2014), depending on the data availability in the Pakistan Microfinance Reviews.

### 3.2 The input/output variables

The estimates obtained from Data Envelopment Analysis (DEA) largely depend on input and output variables selection. In turn, the input and output variables selection depends on the approach adopted for assessing financial institutions. There are three approaches, namely production, intermediation, and assets. The production approach views financial institutions as productive units or firms. The intermediate approach considers financial institutions as a mediator between an investor and savers, while in the assets approach, outputs are determined through assets and productivity of advances [27]. Since MFPs are mostly non-regulated in Pakistan, and deposits as an input variable are missing, the current study uses the production approach for the analysis of the social and financial efficiency of the POs. The input and output variables are described in Table 1.

**Table 1:** Description of input and output variables.

Symbol	Variable name	Variable definition	Unit
Input (a)	Total Assets	Total of all net asset accounts	PKR
Input (b)	Operating Cost	Expenses related to operations include personnel expenses, rent and utilities, transportation, office supplies, and depreciation.	PKR
Input (c)	Number of loan officers	The number of individuals the Microfinance Providers (MFPs) actively employ to disburse the loan and collect repayments.	Number
Output (1)	Financial revenue	Financial revenue generated from the gross loan portfolio and investments plus other operating revenue	PKR
Output (2)	Outreach to poor clients	The number of active borrowers	Number
Output (3)	Number of women borrowers	The number of active female borrowers	Number

Institutional characteristics, along with assets & cost structures, are represented with the help of input variables, while social and financial aspects of the POs are expressed through output variables, including financial revenue, outreach, and female borrowers (Gutiérrez et al., 2009; Gutierrez and Lezama, 2011) [28, 29]. Analysts suggest the aspects of risks and efficiency to evaluate the performance of financial institutions [20]. The current study has employed the aspect of efficiency, but not risk, to investigate the performance of the POs of PPAF.

According to Green [30], "efficiency is the degree to which an action reaches its maximum output with minimum use of resources to achieve certain goals". The relationship between the input and output variables is established with the help of production process blocks. The blocks comprise estimation techniques, operational scale, and managerial & technical know-how. Those POs/MFPs are connected through the input and output approach that meet the requirements of the microfinance sector.

### 3.3 Analytical technique

The performance of MFPs can generally be measured with the help of various financial ratios. These ratios are simple to compute and easy to interpret, but these provide incomplete information and may lead to misleading conclusions. Moreover, efficiency (e.g., technical efficiency) is a multidimensional concept and generally involves many inputs and outputs [31–33]. Therefore, it is generally not recommended to measure the efficiency with the help of financial ratios [20]. Alternatives of the financial ratios to measure efficiency includes Distribution Free Approach (DFA), Free Disposal Hull (FDU), Stochastic Frontier Analysis (SFA), Thick Frontier Approach (TFA), Mixed Optimal Strategy (MOS), Index Number and DEA [34].

Amongst these, the use of DEA for the performance evaluation of financial institutions is generally the preferred method [34–37]. DEA produces good results even in small samples and does not need price information [38]. The techniques also require no assumption about the distribution of the variables and can be used for several inputs and outputs in the model [28, 29, 38–40]. Owing to these advantages against other competing methods, the majority of the empirical studies use DEA for performance evaluation of the MFPs (see for instance, [38, 40–51]. Following the bulk of the literature, the current study also uses DEA to examine the social and financial efficiency of the POs operating under PPAF.

To formally outline the method, let *Y*_*ik*_ denote the *i*^*th*^ output produced by the *k*^*th*^ MFP, *X*_*jk*_ be the *j*^*th*^ input used by the *k*^*th*^ MFP, *V*_*j*_ and *U*_*i*_ be the weights assigned, respectively, to the *j*^*th*^ input and *i*^*th*^ output. Assuming *m* outputs and *n* inputs, technical efficiency (denoted by *θ*_*k*_) of the *k*^*th*^ MFP can be expressed as [52–55]

$$\theta_{k}=\frac{\sum_{i=1}^{m}U_{i}Y_{ik}}{\sum_{j=1}^{n}V_{j}X_{jk}}$$ (1)

Thus, technical efficiency is the ratio of sum of the weighted output to sum of the weighted inputs of the *k*^*th*^ MFP. The kth MFP, also known as Decision Making Unit (DMU), maximizes Eq (1), subject to the following constraint.


(∑i=1muiyik∑j=1n.vjxjk)≤1,whereujandvj≥0 (2)

The constraint in Eq (2) states that the efficiency value of the *k*^*th*^ MFPs must be less than one, along with positive input & output weights. Weight is chosen so that the DMUs should maximize their respective efficiencies. The following output-oriented mathematical programming is used for the selection of optimal weights.


$$\begin{array}{l} \mathrm{Max}.\mathrm{TE}(\theta )=\sum_{i=1}^{m}u_{i}y_{iko}\\ Subject\;to\\ \sum_{i=1}^{m}u_{i}y_{\mathrm{ik}}-\sum_{j=1}^{n}v_{j}X_{\mathrm{jk}}\leq 1\\ \sum_{j=1}^{n}v_{j}x_{jo}=1 \end{array}$$ (3)

Eq (3) can be interpreted as follow;

It maximizes the weighted sum of outputs i for the Ko DMU. "A constraint is used that the weighted sum of input j for k^th^ MFP is subtracted from the weighted sum of output i, for k^th^ MFP that is less than or equal to one [52–53, 55].

The above-mentioned dual problem is constructed as;

$$\begin{array}{l} \mathrm{Min}\;\theta =\theta o-\varepsilon (\sum_{i=1}^{m}s_{i}{}^{+}+\sum_{j=1}^{n}s_{j}{}^{-})\\ \mathrm{Subject}\;\mathrm{to}\\ \sum_{j=1}^{n}x_{ik}\lambda_{k}+s_{j}{}^{-}=\theta_{x_{io},}\;j=1,\ldots ..n\\ \sum_{j=1}^{m}y_{jk}\lambda_{k}-s_{i}{}^{+}=y_{x_{io}},\;i=1,\ldots ..m\\ \lambda_{j},s_{j}{}^{-},s_{i}{}^{+}\geq 0,\varepsilon >0,k=1,\ldots \ldots ..s \end{array}$$ (4)

Eq (4) can be explained as follow:

Sign *θo* indicates the input size of the DMU’s, which is required to produce an output quantity equivalent to its benchmarked DMU, weighted by the λi. Slack variables; "the variables that are defined to transform an inequality expression into an equality expression with an added slack variable. The slack variable is defined by setting a lower bound of zero (>0)". A slack variable is helpful in transforming inequality constraint into non-negativity or equality constraint [56]. Si-Sj+ indicates input & outputs. λ_j_ specifies- n multiplying by one, which the column vector of constant & denotes benchmarked DMUs. Charnes et al. [57] developed the input-oriented (CCRI) model. Their model is based on the Constant Return to Scale (CRS) to estimate the efficiency of the DMUs. CCRI model is used in the current study to estimate the Overall Technical Efficiency (OTE) of the DMUs. Pure Techincal and Scale Efficiencies are assumed to be assessed with the help of OTE (because OTE has both types of efficiencies). Still, the assumption may not be valid in imperfect competitions. Banker et al. [58] modified the CCRI model, later known as the Banker, Charnes & Cooper input-oriented (BCCI) model. Banker, Charnes & Coper (BCC) model has the advantage of measuring Pure Technical Efficiency (PTE). PTE indicates the managerial & technical efficiency of the DMUs. The efficiencies of the MFPs are estimated through CCRI & BCCI models to capture a more holistic picture of social & financial performance. The advanced form of the CCR model is as follows:

$$\begin{array}{l} \mathrm{Min}\;\mathrm{TE}(\theta )=\theta o-\varepsilon (\sum_{i=1}^{m}s_{i}{}^{+}+\sum_{j=1}^{n}s_{j}{}^{-})\\ \;\mathrm{Subject}\;\mathrm{to}\\ \;\sum_{j=1}^{n}x_{ik}\lambda_{k}+s_{j}{}^{-}=\theta_{x_{io}},\;j=1,\ldots ..n\\ \;\sum_{j=1}^{m}y_{jk}\lambda_{k}-s_{i}{}^{+}=y_{x_{io,}}\;i=1,\ldots ..m\\ \;\sum_{k=1}^{s}\lambda_{k}=1\\ \;\lambda_{j},s_{j}{}^{-},s_{i}{}^{+}\geq 0,\varepsilon >0,k=1,\ldots \ldots .n \end{array}$$ (5)

The social and financial efficiency of the POs are investigated by assuming Constant Return to Scale (Overall Technical Efficiency), Variable Return to Scale (Pure Technical Efficiency), and the operational level of the POs (Scale Efficiency). The study aims to assess the managerial efficiency of the POs for the generation of financial revenue as well as outreach to poor clients (social efficiency). Moreover, the sources of social and financial inefficiency (if any) have been assessed through the estimation of PTE and Scale Efficiency (ScE.).

To draw meaning full results, the data for the current study is treated as follows;

- The year-wise percentage average values have been estimated to assess trends in the social & financial efficiency of the POs.
- Mean average, an average of the percentage average social & financial efficiency values, have been estimated to understand the social & financial efficiency of the POs over the study period. (Year-wise social & financial efficiency scores are summarized to a single value).

### 3.4 Operational self-sufficiency

Micro Finance Providers (MFPs) gradually recognized that microfinance services’ continuous provision/flow requires sustainability. It refers to the covering/fulfillment of not only all types of costs, such as operational, financial & administrative, but also providing enough revenue/profit for the financing of day-to-day operations without depending on the perpetual financial support in the shape of government subsidies and donors funds [59]. According to Brau and Woller [60] the terms self-sufficiency & sustainability are used interchangeably in the standard literature on microfinance. Sustainability is inevitable for financial institutions because the poor demand financial services regularly [61]. Navajas et al. [15] also argue that unsustainable MFPs cannot help the poor continuously because they generally disappear in the long run. In the field of microfinance, profitability and sustainability/self-sufficiency are usually measured with the help of accounting-based indicators such as; OSS, Financial Self-Sufficiency (FSS), Returns on Equity (ROE), and Returns on Assets (ROA) [46, 62–68]. These indicators are also helpful in investigating the financial viability of the MFPs [69, pg.49].

For financial sustainability, the current study has accounted OSS ratio, which is also used in numerous other studies[54, 70–76]. OSS measures how MFP utilizes its sufficient revenue to absorb the total costs, i.e. operating costs, loan loss provisions and financial costs, irrespective of subsidies, grants & donations. The sustainability of MFPs starts with OSS, where the operating costs of the institutions are covered regardless of the revenue sources [73]. The expression used to calculate OSS ratio can be described as follow [68]:

$$OSS\frac{Total\;Operating\;Revenue}{Financial\;expenses+operational\;costs+loss\;on\;loan\;expenses}$$

Where if;

OSS < 100% = Unsustainable

100% < OSS < 110% = Operationally Sustainable

OSS > 100% = Financial Sustainability.

## 4 Results and discussions

### 4.1 Social and financial efficiency analyses of the POs assuming constant returns to scale

The year-wise average social and financial efficiencies under CRS (Overall Technical Efficiency) lie from 64.05% to 84.37% and from 41.81% to 83.97%, respectively. Alternatively, subtracting these values from 100, social inefficiency ranges from 15.63% to 35.95%, and financial inefficiency ranges from 16.03% to 58.19%. The social and financial inefficiency range reflects the managerial inefficiency of the POs during the study period. The mean average social and financial efficiency of the POs under CRS is 73.37% (26.63% inefficiency) and 74.00% (26% inefficiency), respectively, which suggests that the managerial efficiency of the POs is 73.37% to reach poor clients (social efficiency) and 74.00% to generate financial revenue (financial efficiency) over the entire study period. Table 2 shows measured/ estimated values of the social and financial efficiencies expressed as the percentage average scores of the POs measured under CRS. To assess the sources of inefficiencies, the social & financial efficiency of the POs are gauged by assuming Variable Return to Scale (VRS) and OS / ScE.

**Table 2:** Overall technical efficiency of partner organizations, operating under Pakistan Poverty Alleviation Fund (PPAF).

	No of Decision-Making Units	Number of Efficient DMUs (Under Constant Return Scale)		Averages of Efficiency Scores (Under Constant Return Scale)		Mean Averages of Efficiency Scores (Under Constant Return Scale)	
years		Socially Efficient	Financially Efficient	Social Efficiency	Financial Efficiency	Social	Financial
2005	14	8	5	79.14	78.38	1107.83	1097.20
2006	15	7	4	78.04	76.11	1170.46	1141.50
2007	14	6	3	84.38	75.11	1181.19	1050.15
2008	19	6	7	77.82	80.27	1478.38	1525.33
2009	19	4	4	77.17	80.83	1466.05	1535.97
2010	19	4	7	75.10	83.96	1426.90	1595.44
2011	20	10	4	80.70	83.77	1614.00	1675.60
2012	30	6	1	65.97	41.82	1978.80	1254.30
2013	33	6	8	64.10	79.70	2113.66	2628.50
2014	35	6	5	68.78	73.20	2406.96	2561.30
2015	27	7	5	75.10	76.53	2029.07	2066.05
Total	245					17973.24	18131.23
Mean Average						73.37	74.00

### 4.2 The efficiency analyses of the POs assuming variable returns to scale (VRS)

The percentage average of social and financial efficiency scores of POs under VRS (PTE) lies in in the range from 77.38% to 93.78% (range of social inefficiency from 6.22% to 22.62%) and from 71.88% to 93.72% (range of financial inefficiency from 6.28% to 28.12%), respectively. A slight difference can be observed between the yearly social & financial efficiencies over time. Such trends in the average efficiency scores are also revealed by (Crawford et al. [77]). The mean average social and financial efficiency scores under VRS (PTE) are 83.17% (16.82% inefficiency) and 84.27% (15.73% inefficiency), respectively, which suggests that the microcredit program of the POs are 83.17% efficient regarding revenue generation and 84.27% efficient to reach the poor client during the entire study period. The results further suggest that the POs can produce the same social & financial output level by decreasing the inputs by 16.81% and 15.72%, respectively. Table 3 shows the yearly percentage average of the social and financial efficiency of the POs measured under VRS (PTE).

**Table 3:** Pure technical efficiency (VRS) of partner organizations operating under the Pakistan Poverty Alleviation Fund (PPAF).

	No of Decision Making Units	Number of Efficient DMUs		Mean Averages of Efficiency Scores		Mean Averages of Efficiency Scores	
years		Social	Financial	Social	Financial	Social	Financial
2005	14	10	7	87.53	85.57	1225.27	1197.85
2006	15	11	6	90.73	84.23	1361.10	1263.30
2007	14	9	8	93.79	86.33	1312.93	1208.75
2008	19	12	8	87.67	84.34	1665.91	1602.66
2009	19	7	7	84.14	85.90	1598.42	1632.10
2010	19	9	12	84.32	93.73	1602.28	1780.69
2011	20	12	7	85.29	88.83	1705.60	1776.80
2012	30	13	12	76.91	71.89	2307.60	2156.40
2013	33	14	14	77.39	84.68	2553.55	2794.76
2014	35	14	12	80.82	84.57	2828.34	2959.60
2015	27	13	9	82.20	84.36	2219.68	2277.44
Total	245					20380.73	20650.36
Mean Average						83.17	84.27

Source: Researches Own Calculation

### 4.3 Efficiency analyses of the pos concerning the operational scale

The yearly percentage average social & financial efficiency scores measured concerning the OS of the POs lie in the range of 83.48% & 94.15% (range of social inefficiency 5.85% & 16.52%) and 87% & 94.38% (range of financial inefficiency 5.62% & 13%), respectively, over time. The mean average social & financial efficiency scores measured with respect to the OS are 87.67% (12.33% social inefficiency) and 88.03% (11.97% financial inefficiency), respectively. The results further reveal over the entire study period that the POs are 87.67% close to optimal scale concerning outreach and 88.03% close to revenue generation. Table 4 shows the estimated percentage average social & financial efficiency scores concerning the OS of the POs.

**Table 4:** Scale efficiency of partner organizations under the Pakistan Poverty Alleviation Fund (PPAF) with respect to the operational scale.

	No of Decision Making Units	Number of Efficient DMUs (With Respect to Scale)		Averages of Efficiency Scores (With Respect to Scale)		Mean Averages of Efficiency Scores (With Respect to Scale)	
years		Financial (Scale Efficiency)	Social (Scale Efficiency)	Social (Scale Efficiency)	Financial (Scale Efficiency)	Social (Scale Efficiency)	Financial (Scale Efficiency)
2005	14	9	9	89.06	92.57	1246.70	1296.13
2006	15	8	7	84.27	90.36	1263.90	1355.24
2007	14	4	7	88.98	87.00	1245.57	1218.00
2008	19	11	7	87.02	94.81	1653.57	1801.57
2009	19	6	4	91.18	94.11	1732.61	1787.90
2010	19	8	5	89.05	88.98	1691.77	1690.44
2011	20	10	12	94.16	94.31	1883.00	1886.40
2012	30	4	8	85.83	64.16	2574.61	1924.50
2013	33	14	7	83.47	94.37	2754.85	3114.55
2014	35	7	9	85.26	86.78	2984.46	3036.96
2015	27	8	11	90.80	91.09	2451.34	2459.17
Total	245					21482.35	21570.84
Average of Average						87.67	88.03

After comparing the efficiency scores (these results consistent to Anwar *et al*, 2021) measured under various scales, it can be concluded that the inefficiency of the POs is mainly because of the miss-utilization of resources rather than the improper scale of operations.

### 4.4 Shifting emphasis from social uplift to revenue generation by POs

Analyses of the trends in the percentage average social & financial efficiency scores (estimated under CRS & VRS) reflect the shifting emphasis of the POs from the social uplift of the poor masses to more revenue generation. During the initial years of the study, i.e. from 2005 up to almost 2008, the POs are realized more motivated towards the social uplift of the poor masses. Over time the motivation shifted towards revenue generation. These findings are also consistent with study [77], they further concluded that social & financial efficiencies need not be mutually exclusive. It is also revealed that despite the rising number of POs over time, less improvement can be observed in the POs of PPAF.

### 4.5 Comparison of the efficiency scores

Table 5 summarizes and compares the mean average social & financial efficiency scores measured under CRS, VRS, and OSof the POs. The higher mean average social & financial efficiencies of the POs under the assumption of the scale of operation (see Table 5) indicates that the POs operate closer to the optimal scale (scale efficient) than the utilization of the inputs (Pure Technically Efficient). It is further noticed that the inefficiency is because of twin problems, i.e., wastage of resources and sub-optimal scale of operations. Still, the sub-optimal scale of operations may not be considered a severe issue in the short run. Bassem [78, 79] also confirms these results; Kipesha [80]; and Tahir & Tahrim [81]. It is further revealed from Table 5 that the mean average efficiency scores improved when the assumption of CRS is relaxed.

**Table 5:** Comparison of efficiency scores of the partner organizations.

Mean Average Efficiency	Overall Technical Efficiency (CRS)	Pure Technical Efficiency (VRS)	Scale Efficiency (ScE)
**Financial Efficiency**	74.00%	84.27%	88.04%
**Social Efficiency**	73.36%	83.16%	87.67%

Table 6 explains the percentage average sustainability status of the POs for 2005–2015. The POs such as ASA-P, GBTI, OPP / OCT, SDF, and Sungei have a high sustainability value of equal to or more than 150%. About 77.5% of the POs are noticed as self-sustainable over the entire study period, i.e. 2005–2015. With a maximum score of 177.49%, ASA-P has noticed a highly self-sufficient PO of PPAF.

**Table 6:** Average suitability status of Pakistan Poverty Alleviation Fund’s partner organizations.

S.No.	DMUs / POs	Average Self-Sufficiency of the Pos	Average Sustainability Status of the POs
1	ASA-P	177.49%	**Sustainable**
2	Akhuwat	95.49%	Unsustainable
3	Agahe	111.33%	**Sustainable**
4	AMRDO	110.40%	**Sustainable**
5	Asasah	65.93%	Unsustainable
6	BRAC-P	92.23%	Unsustainable
7	BEDF	100.00%	**Sustainable**
8	BAIDARIE	84.05%	Unsustainable
9	CSC	94.55%	Unsustainable
10	CWCD/ Wasil	84.20%	Unsustainable
11	DAMEN	105.37%	**Sustainable**
12	FFO	100.00%	**Sustainable**
13	GBTI	149.60%	**Sustainable**
14	IRP	107.60%	**Sustainable**
15	JWS	116.69%	**Sustainable**
16	Kashf	115.88%	**Sustainable**
17	Mojaz	105.87%	**Sustainable**
18	NRDP	132.05%	**Sustainable**
19	OPP / OCT	150.57%	**Sustainable**
20	OPD	108.95%	**Sustainable**
21	ORIX / OLP	126.62%	**Sustainable**
22	RCDS	146.77%	**Sustainable**
23	SAATH	139.33%	**Sustainable**
24	SDS	123.40%	**Sustainable**
25	SRDO	106.70%	**Sustainable**
26	SVDP	122.98%	**Sustainable**
27	SSSWA	102.70%	**Sustainable**
28	SAFWCO	100.89%	**Sustainable**
29	SDF	172.55%	**Sustainable**
30	SUNGI	173.98%	**Sustainable**
31	TF	28.00%	Unsustainable
32	VDO	114.80%	**Sustainable**
33	NRSP	120.90%	**Sustainable**
34	PRSP	138.89%	**Sustainable**
35	SRSP	93.51%	Unsustainable
36	TRDP	104.30%	**Sustainable**
37	SRSO	102.27%	**Sustainable**
38	KB	108.74%	**Sustainable**
39	NMFBL	78.83%	Unsustainable
40	NRSPBL	118.70%	**Sustainable**

Table 7 presents the percentage average financial, social and overall efficiency scores of each POs measured under the assumption of CRS, VRS & OS. The average OTE, PTE, and ScE estimated under the input-oriented model are 69.73%, 82.09%, and 85.05%, respectively. The POs can collectively reduce their inputs by 16.20% without any effects on the current level of output, i.e. revenue generation and outreach to poor clients. Moreover, Pure Technical Inefficiency is noticed slightly more than Scale Inefficiency. It implies that Overall Technical Inefficiency is primarily because of miss utilization of resources i.e, managerial inefficiency (Pure Technical Inefficiency) rather than the improper scale of operation, i.e. (Scale Inefficiency). The results further indicate that under input-oriented DEA specification, 72.55% of the POs are experiencing economies of scale, and 22.5% of the POs are at the Decreasing Return to Scale stage. These results are also consistent with the results of study [54].

**Table 7:** Average financial and social efficiency scores of the POs under constant returns to scale (CRS), variable return to scale (VRS) and with respect to operational scale.

.No.	DMUs / POs	Average Efficiency Scores under Constant Returns to Scale (CRS) (2005–15)		Mean Average Overall Efficiency Scores	Average Efficiency Scores under Variable Returns to Scale (VRS) (2005–15)		Mean Average Overall Efficiency Scores	Average Efficiency Scores with Respect to Operational Scale (OS) (2005–15)		Mean Average Overall Efficiency Scores	RTS
		Financial Efficiency	Social Efficiency		Financial Efficiency	Social Efficiency		Financial Efficiency	Social Efficiency		
1	ASA-P	84.72%	99.43%	92.08%	90.47%	100.00%	95.24%	94.07%	99.43%	96.75%	IRS
2	Akhuwat	44.95%	91.80%	68.37%	59.46%	98.26%	78.86%	79.21%	94.38%	86.79%	IRS
3	Agahe	52.34%	81.42%	66.88%	68.03%	90.01%	79.02%	78.62%	90.77%	84.69%	IRS
4	AMRDO	55.33%	77.58%	66.46%	61.14%	78.64%	69.89%	87.75%	98.59%	93.17%	IRS
5	Asasah	81.20%	83.84%	82.52%	82.42%	87.48%	84.95%	98.60%	95.57%	97.08%	IRS
6	BRAC-P	83.43%	60.24%	71.84%	91.21%	65.41%	78.31%	91.66%	91.79%	91.72%	IRS
7	BEDF	80.55%	65.91%	73.23%	100.00%	100.00%	100.00%	80.55%	65.91%	73.23%	DRS
8	BAIDARIE	49.17%	39.26%	44.21%	72.70%	66.67%	69.68%	66.74%	59.47%	63.11%	DRS
9	CSC	75.86%	63.84%	69.85%	79.33%	65.85%	72.59%	95.14%	96.51%	95.82%	IRS
10	CWCD/ Wasil	87.25%	42.58%	64.91%	88.29%	46.72%	67.50%	98.64%	90.61%	94.63%	IRS
11	DAMEN	88.60%	88.53%	88.57%	93.62%	91.55%	92.58%	94.27%	96.29%	95.28%	IRS
12	FFO	57.19%	76.41%	66.80%	59.89%	80.25%	70.07%	95.32%	95.25%	95.28%	IRS
13	GBTI	79.28%	68.84%	74.06%	84.39%	77.65%	81.02%	91.92%	85.90%	88.91%	IRS
14	IRP	25.16%	43.34%	34.25%	47.03%	62.00%	54.52%	53.50%	69.90%	61.70%	DRS
15	JWS	70.94%	69.92%	70.43%	76.36%	74.67%	75.52%	93.82%	94.11%	93.97%	IRS
16	Kashf	87.14%	82.98%	85.06%	99.10%	88.63%	93.87%	87.80%	93.01%	90.41%	IRS
17	Mojaz	76.38%	45.06%	60.72%	76.42%	56.28%	66.35%	99.93%	80.73%	90.33%	IRS
18	NRDP	54.70%	40.81%	47.75%	75.43%	56.88%	66.15%	68.88%	72.20%	70.54%	IRS
19	OPP / OCT	84.16%	100.00%	92.08%	85.53%	100.00%	92.76%	99.23%	100.00%	99.62%	IRS
20	OPD	64.99%	65.04%	65.01%	75.65%	69.67%	72.66%	84.29%	93.24%	88.76%	IRS
21	ORIX / OLP	82.64%	94.99%	88.82%	87.63%	96.33%	91.98%	94.38%	98.48%	96.43%	IRS
22	RCDS	76.79%	70.22%	73.50%	81.24%	73.25%	77.24%	93.95%	95.42%	94.68%	IRS
23	SAATH	81.80%	61.50%	71.65%	92.53%	78.48%	85.50%	88.06%	79.88%	83.97%	DRS
24	SDS	21.71%	78.92%	50.32%	100.00%	100.00%	100.00%	21.71%	78.92%	50.32%	DRS
25	SRDO	59.09%	46.92%	53.01%	93.41%	90.15%	91.78%	64.67%	55.99%	60.33%	DRS
26	SVDP	71.00%	43.22%	57.11%	76.68%	48.47%	62.57%	89.02%	88.88%	88.95%	IRS
27	SSSWA	71.74%	100.00%	85.87%	100.00%	100.00%	100.00%	71.74%	100.00%	85.87%	CRS
28	SAFWCO	72.56%	86.28%	79.42%	74.72%	88.34%	81.53%	97.14%	97.49%	97.32%	IRS
29	SDF	78.81%	100.00%	89.41%	100.00%	100.00%	100.00%	78.81%	100.00%	89.41%	CRS
30	SUNGI	74.08%	81.86%	77.97%	90.77%	100.00%	95.38%	81.33%	95.26%	88.30%	DRS
31	TF	61.37%	48.77%	55.07%	61.49%	49.51%	55.50%	99.80%	98.51%	99.16%	IRS
32	VDO	52.64%	76.02%	64.33%	99.78%	100.00%	99.89%	52.70%	76.02%	64.36%	DRS
33	NRSP	77.15%	67.50%	72.33%	94.68%	100.00%	97.34%	81.95%	67.50%	74.72%	IRS
34	PRSP	76.73%	77.06%	76.89%	83.09%	81.56%	82.32%	93.25%	92.71%	92.98%	IRS
35	SRSP	81.71%	87.72%	84.71%	99.48%	100.00%	99.74%	82.14%	87.72%	84.93%	DRS
36	TRDP	77.45%	84.93%	81.19%	82.82%	90.14%	86.48%	93.06%	94.23%	93.65%	IRS
37	SRSO	82.06%	93.48%	87.77%	89.00%	94.48%	91.74%	92.55%	98.51%	95.53%	IRS
38	KB	81.68%	60.70%	71.19%	97.21%	97.36%	97.29%	84.41%	62.76%	73.58%	IRS
39	NMFBL	49.95%	14.06%	32.01%	65.05%	32.85%	48.95%	86.88%	62.06%	74.47%	IRS
40	NRSPBL	78.25%	31.38%	54.81%	96.74%	53.55%	75.14%	67.57%	59.33%	63.45%	IRS
	Average	69.81%	69.81%	69.81%	83.32%	80.78%	82.05%	83.88%	86.33%	85.11%	

**Note:** RTS stands for Rate of Returns Scale, IRS stands for Increasing Returns to Scale, CRS stands for Constant Returns to Scale, DRS stands for Decreasing Returns to Scale.

## 5 Conclusion and policy recommendations

Efficiency assessment of the MFPs is indispensable for all stakeholders to ensure optimal policy agenda. The financial efficiency of the MFPs is essential as, in the absence of grants and donations, outreach also depends on it. Therefore, this paper focuses on assessing the financial and social efficiency of the Partner Organizations (POs) of the PPAF. An unbalanced panel for 2005–2015 is constructed, collecting relevant data from Pakistan Microfinance Network. The framework of DEA, a nonparametric and non-stochastic linear programming-based efficiency analysis technique, is applied with three input and three output variables. The average financial and social efficiency has been checked in the light of CRS, VRS, and concerning OS of the POs. Trends in the average efficiency scores revealed the shifting emphasis of the POs from performing their social role to more income/profit generation.

The mean average efficiency scores are estimated to evaluate the efficiency of the POs over the entire study period. The mean average financial and social efficiency scores, under CRS and VRS, reveal social efficiency to be greater than financial efficiency during 2005–2008. The trend, however, reversed during 2008–2015, with only one exception in 2012. The shock in 2012 is usually attributed to the instability and structural deficits, during which the financial markets suffered from limited resource bases and deposits [82]. It shows that POs of the PPAF remained more motivated toward financial revenue than the social uplift of the poor masses. This finding is in line with prior research, which attributes the shifting emphasis of the MFPs to capital market penetration and commercialization of the microfinance sector [69].

Moreover, donors and governments have gradually withdrawn funds from the microfinance sector to promote a sense of self-reliance in the industry [51]. But given the primary objective of MFPs–to serve the otherwise un-bankable segments of society–this shift in emphasis may be counter-productive. Financial sustainability becoming the sole objective of MFPs would essentially imply no difference in conventional financial institutions and microfinance, and the riskier (deprived) segments of society would be the first to bear the burden of such a development. Hence, it is recommended that the donor agencies and governments may continue with conditional grants tied to the social efficiency levels of the MFPs.

The OTE has been decomposed into PTE and ScE to understand the sources of the inefficiency. Pure Technical Inefficiency is revealed as a significant source of inefficiency in the short run. Results of the study further indicate that 77.5% of the POs were self-sustainable over the study period. Since the same social and financial efficiency levels can be achieved by reducing approximately 16% of the inputs, it is concluded that resources are wasted. Hence, the study recommends objective-oriented training and workshops for the management and staff of MFPs to overcome the problem of miss-utilization of resources.

## 6 Limitations of the study

The study’s main limitation includes the number of MFPs covered in the sample and the use of an unbalanced panel for efficiency assessment. As mentioned earlier, the total number of MFPs under the umbrella of PPAF is more than 130. The current study, however, covers only 35 of the POs. Both these limitations were due to data unavailability; hence, future research needs to carry out efficiency assessment on a more significant number of POs, covering more years.

## Supporting information

S1 Data (XLSX)

S1 File (DOCX)
