# Supplementary material for: Financial and social efficiency of microcredit programs of partner organizations of Pakistan Poverty Alleviation Fund
Source: PLoS One. 2023 Mar 24;18(3):e0280731. doi: 10.1371/journal.pone.0280731 (PMC10038267; doi:10.1371/journal.pone.0280731)
Supplement: S1 File — (DOCX) [file pone.0280731.s002.docx]

| **S.No.** | **DMU Abbreviation** | **DMU Full & Establishment Date** | **Type** | **Location** |
| --- | --- | --- | --- | --- |
| 1 | ASA-P | Association for Social Advancement- Pakistan, 2008 | Non-profit,self-sufficient microfinance institution  Regulated | International/Bangladesh Based |
| 2 | Akhuwat | Akhuwat, 2001 | NGO/Not for Profit/Nonregulated | International / Pakistan Based |
| 3 | Agahe | [Association for Gender Awareness & Human Empowerment, 2001](https://pakngos.com.pk/listing/association-for-gender-awareness-human-empowerment-agahe/) | NGO/Non Profit/ Regulated | Local/ Pakistan Based |
| 4 | AMRDO | [Al-Mehran Rural development organization. (1998)](http://www.amrdo.org/) | NGO/ Non Profit/ Regulated | Local/ Pakistan Based |
| 5 | Asasah | Asasah (2003) | MFI/ Non Profit/ Nonregulated | Local/ Pakistan Based |
| 6 | BRAC-P | Bangladesh Rural Advancement Committee – Pakistan, 2007 | NGO/Not for Profit/ Regulated | International/Bangladesh Based |
| 7 | BEDF | [Badbaan Enterprise Development Forum](https://www.mftransparency.org/microfinance-pricing/pakistan/033-BEDF/), 2006. | NGO/ Non Profit/ Nonregulated | Local/ Pakistan Based |
| 8 | BAIDARIE | BAIDARIE ,1993 | NGO/ Non Profit/ Nonregulated | Local/ Pakistan Based |
| 9 | CSC | Community Support Concern (CSC), 1989 | NGO/ Non Profit/ Nonregulated | Local/ Pakistan Based |
| 10 | CWCD/ Wasil | [Centre for Women Co-Operative Development](https://www.devex.com/organizations/centre-for-women-co-operative-development-46798)  or Wasil, (2002) | Other/NGO/ Non Profit/ Unregulated | Local/ Pakistan Based |
| 11 | DAMEN | [Development Action for Mobilization and Emancipation, 1992 (DAMEN)](http://www.damen-pk.org/) | NGO/ Non Profit/ Nonregulated | Local/ Pakistan Based |
| 12 | FFO | Farmers Friends Organization (FFO),2003 | NGO(Non-Profit), Non-Regulated | Local/ Pakistan Based |
| 13 | GBTI | [Ghazi Barotha Taraqiati Idara](https://gbti.org.pk/), (1995) | NGO/ Non Profit/ Nonregulated | Local/ Pakistan Based |
| 14 | IRP | Islamic Relief Pakistan, 1984 | NGO/ Not for Profit/ Nonregulated | International/ UK Based |
| 15 | JWS | [Jinnah Welfare Society, (1992).](https://www.jws.org.pk/) | NGO/ Not-for-Profit/ Nonregulated | Local/ Pakistan Based |
| 16 | Kashf (KF) | [Kashf Foundation, 1996](https://kashf.org/) | NGO/ Not-for-Profit/ Nonregulated | Local/ Pakistan Based |
| 17 | Mojaz | Mojaz Foundation, 2008. | NGO/Not-for-Profit | Local/ Pakistan Based |
| 18 | NRDP | [National Rural Development Program](https://www.mftransparency.org/microfinance-pricing/pakistan/043-NRDP/). (1992). | NGO/ Not-for-Profit/ Regulated | Local/ Pakistan Based |
| 19 | OPP / OCT | [Orangi Pilot Project](http://www.opp.org.pk/) /[Orangi Charitable Trust](https://oprct.org/), 1989. | NGO/ Not-for-Profit/ Unregulated | Local/ Pakistan Based |
| 20 | OPD | [Organization For Participatory Development](https://www.mftransparency.org/microfinance-pricing/pakistan/044-OPD/), 1991 | NGO/ Not-for-Profit/ Unregulated | Local/ Pakistan Based |
| 21 | ORIX / OLP | [Orix Leasing Pakistan Ltd](https://www.mftransparency.org/microfinance-pricing/pakistan/017-OLP/). 1986 | [Non-Bank Financial Institution](https://www.mftransparency.org/glossary#Non-Bank-Financial-Institution) (NBFI)/  Regulated. | Local/ Pakistan Based |
| 22 | RCDS | [Rural Community Development Society](https://www.mftransparency.org/microfinance-pricing/pakistan/021-RCDS/), 1998. | NGO/ Not-for-Profit / Regulated. | Local/ Pakistan Based. |
| 23 | SAATH | Saath Microfinance Foundation Pakistan, 2006 | Non-Bank Financial Company. (NBFC)/For Profit/Unregulated | Local/ Pakistan Based. |
| 24 | SDS | SAATH Development Society, 2003 | NGO/ Not-for-Profit./ Unregulated | Local/ Pakistan Based. |
| 25 | SRDO | [Shadab Rural Development Organization](https://www.mftransparency.org/microfinance-pricing/pakistan/048-SRDO/), 2000 | NGO/ Not-for-Profit/ Regulated. | Local/ Pakistan Based. |
| 26 | SVDP | [Soon Valley Development Program](https://www.mftransparency.org/microfinance-pricing/pakistan/050-SVDP/), 1996 | NGO/ Not-for-Profit/ Unregulated | Local/ Pakistan Based. |
| 27 | SSSWA | Shah Sachal Sami welfare Association, 1996. | NGO/ Not-for-Profit/  Voluntary Social Welfare Organization | Local/ Pakistan Based. |
| 28 | SAFWCO | Sindh Agricultural & Forestry Workers coordinating organization. 1986 | NGO/ Not-for-Profit/ Unregulated | Local/ Pakistan Based. |
| 29 | SDF | Salik Development Foundation, 1989 | NGO/ Not-for-Profit/ Unregulated | Local/ Pakistan Based. |
| 30 | SUNGI | [Sungi Development Foundation](https://www.mftransparency.org/microfinance-pricing/pakistan/025-SDF/), 1990 | NGO/ Not-for-Profit/ Unregulated | Local/ Pakistan Based. |
| 31 | TF | Taraqee Foundation, 1994. | NGO/ Not-for-Profit/ Unregulated | Local/ Pakistan Based. |
| 32 | VDO | [Villagers Development Organization](https://www.mftransparency.org/microfinance-pricing/pakistan/052-VDO/), 1993 | NGO/ Not-for-Profit/ Regulated. | Local/ Pakistan Based. |
| 33 | NRSP | [National Rural Support Program](https://www.mftransparency.org/microfinance-pricing/pakistan/015-NRSP/), 1991 | NGO/ Not-for-Profit/ Nonregulated | Local/ Pakistan Based. |
| 34 | PRSP | [Punjab Rural Support Programme](https://www.mftransparency.org/microfinance-pricing/pakistan/020-PRSP/), 1997. | NGO/ Not-for-Profit/ Regulated. | Local/ Pakistan Based. |
| 35 | SRSP | Sarhad Rural Support Programme, 1989 | NGO/ Not-for-Profit/Regulated | Local/ Pakistan Based. |
| 36 | TRDP | [Thardeep Rural Development Programme](https://www.mftransparency.org/microfinance-pricing/pakistan/027-TRDP/), 1998 | NGO/ Not-for-Profit/ Regulated. | Local/ Pakistan Based. |
| 37 | SRSO | [Sindh Rural Support Organization](https://www.mftransparency.org/microfinance-pricing/pakistan/024-SRSO/), 2003 | NGO/ Not-for-Profit Regulated. | Local/ Pakistan Based. |
| 38 | KBL | [Khushhali Bank Limited](https://www.mftransparency.org/microfinance-pricing/pakistan/013-KBL/), 2000. | Microfinance [Bank](https://www.mftransparency.org/glossary#Bank)/ Regulated. | Local/ Pakistan Based. |
| 39 | NMFBL | Network Microfinance Bank Limited, 2005 | Microfinance [Bank](https://www.mftransparency.org/glossary#Bank)/ Regulated. | Local/ Pakistan Based. |
| 40 | NRSPBL | NRSP Microfinance Bank Ltd, 2011 | Microfinance [Bank](https://www.mftransparency.org/glossary#Bank)/ Regulated | Local/ Pakistan Based. |
|  |  |  |  |  |
|  |  |  |  |  |
